# Draft Genome Sequence of *n*-Alkane-Utilizing Acinetobacter sp. Strain BS1, Isolated from Ethane Oxidation Culture

**DOI:** 10.1128/genomeA.00465-18

**Published:** 2018-05-31

**Authors:** Yong-Wei Yan, Pan-Pan Zhang, Ting Zhu, Zhe-Xue Quan

**Affiliations:** a Key Laboratory for Biodiversity Science and Ecological Engineering, Ministry of Education, Institute of Biodiversity Science, School of Life Sciences, Fudan University, Shanghai, China; b Key Laboratory of Maricultural Organism Disease Control, Ministry of Agriculture and Rural Affairs, Yellow Sea Fisheries Research Institute, Chinese Academy of Fishery Sciences, Qingdao, Shandong, China

## Abstract

Here, we report the draft whole-genome sequence of a bacterial strain, Acinetobacter sp. strain BS1, isolated from black soil during ethane oxidation culture. Medium- or long-chain alkane oxidation-related genes were identified; however, the short-chain alkane monooxygenase was not detected.

## GENOME ANNOUNCEMENT

Microbes of the genus Acinetobacter have been involved in the bioremediation of petroleum (1) because of their high efficiency at degrading different types of alkanes (2). Several pathways and functional genes for the degradation of medium- or long-chain alkanes have been identified for the strains of this genus (3–5). However, the ability to carry out gaseous oxidation has never been reported for these organisms.

To isolate ethane oxidation microbes, a floating-filter method (6) was applied on nitrate mineral salts (NMS) medium (7). Acinetobacter sp. strain BS1 was isolated from colonies on a floating filter through which diluted black soil of the Changbai Mountains in China was filtered and was purified with R2A agar medium. Genomic DNA was extracted using a PowerSoil DNA isolation kit (Mo Bio, USA). The Kapa LTP library kit (Kapa Biosystems, USA) was used to construct the library, and whole-genome sequencing was performed on an Illumina HiSeq 2500 platform using a 2 × 250 protocol. The reads were quality trimmed using Sickle software version 1.33 (8), and bases with a quality above 20 (Q20) were used for assembly by Velvet (version 1.2.10) (9), resulting in 128 scaffolds (including plasmid sequences) with a maximum length of 513 kb and an *N*_50_ value of 138 kb. According to the analysis using CheckM (version 1.0.5) (10), the size of the draft genome was 3,929,328 bp (G+C content, 38.8%), with a coverage of 93.6-fold and completeness of 100%.

Annotation and identification of metabolic pathways were carried out using the NCBI Prokaryotic Genome Annotation Pipeline (PGAP) server. A total of 3,637 coding sequences or open reading frames (ORFs), as well as 64 tRNA genes, were identified. Based on the sequence coverage, about 9 copies of 23S rRNA and 16S rRNA genes were identified, with at least 3 copies of 5S-23S-16S rRNA operons. The draft genome does not encode monooxygenases that play important roles in the oxidation of short-chain (C_1_ to C_4_) *n-*alkanes. However, it encodes a cytochrome P450 hydroxylase that has been reported to be involved in medium-chain *n-*alkane degradation (4). In addition to the genes encoding rubredoxin (*rubA*) and rubredoxin reductase (*rubB*), genes of the AlkB family alkane hydroxylases (11) were also identified and scattered throughout the whole chromosome, which include *alkB* (encoding alkane-1 monooxygenase), *alkJ* (encoding alcohol dehydrogenase), *alkH* (encoding aldehyde dehydrogenase), and *alkK* (encoding acyl-coenzyme A [acyl-CoA] synthetase). Additionally, AlmA- and LadA-encoding genes, involved in long-chain *n-*alkane oxidation, were also identified by the probing of complete protein sequences from Acinetobacter sp. strain DSM 17874 (12) and Geobacillus thermodenitrificans (13), respectively. The utilization of medium- or long-chain alkanes by strain BS1 was confirmed with nonane (C_9_), tridecane (C_13_), or triacontane (C_30_) as a sole carbon source (data not shown).

Strain BS1 can use methanol or ethanol as a sole carbon source but cannot directly use ethane (data not shown). This indicates that strain BS1 may grow during the ethane oxidation process based on the ethanol produced by other ethane-oxidizing microbes, as well as with high contents of methanol-oxidizing bacteria during methane oxidation (14).

### Accession number(s).

This whole-genome shotgun project has been deposited at DDBJ/ENA/GenBank under the accession no. NPMK00000000. The version described in this paper is the first version, NPMK01000000.
